# The impact of visceral adipose tissue as best predictor for difficult colonoscopy and the clinical utility of a long small-caliber scope as rescue

**DOI:** 10.1371/journal.pone.0189817

**Published:** 2017-12-21

**Authors:** Kazuhiro Kashiwagi, Nagamu Inoue, Toshifumi Yoshida, Rieko Bessho, Kazuaki Yoneno, Hiroyuki Imaeda, Haruhiko Ogata, Takanori Kanai, Yoshinori Sugino, Yasushi Iwao

**Affiliations:** 1 Center for Preventive Medicine, Keio University Hospital, Tokyo, Japan; 2 Division of Gastroenterology and Hepatology, Department of Internal Medicine, Keio University School of Medicine, Tokyo, Japan; 3 Department of General Medicine, Saitama Medical University School of Medicine, Saitama, Japan; 4 Center for Diagnostic and Therapeutic Endoscopy, Keio University School of Medicine, Tokyo, Japan; University Hospital Llandough, UNITED KINGDOM

## Abstract

**Background:**

There have been many reports about a variety of factors associated with incomplete colonoscopy or difficult colonoscopy with long cecal intubation time (CIT). The aim of this retrospective study was to analyze the factors related to difficult colonoscopy under conscious sedation and demonstrate the clinical utility of a small-caliber scope as rescue by using the data from a large number of subjects who underwent health check-ups.

**Methods:**

Consecutive 1036 cases over a 12-month period (April 2015 to March 2016) were enrolled and 619 subjects were divided into two groups: Easy colonoscopy (CS) Group (CIT ≤ 10 min); Difficult CS Group (CIT > 10 min or incomplete colonoscopy by a standard scope). The two groups were compared by subjects and colonoscopy characteristics with univariate analysis followed by multivariate logistic regression analysis. Reasons for incomplete colonoscopy were also assessed.

**Results:**

Cecal intubation rate increased from 97.9% to 99.9% (1007/1008) by the rescue scope. Main reasons for incomplete colonoscopy were tortuosity in the left hemicolon (38%), redundancy in the right hemicolon (29%), pain (19%) and fixation (14%). Moreover, 95% (20/21) of rescue colonoscopies were completed without additional sedation. Higher BMI (21 kg/m^2^ ≤ BMI) and intermediate visceral adipose tissue (VAT) (75 cm^2^ ≤ VAT < 150 cm^2^) were significantly associated with easy CS (80.7% vs 19.3%, *P* = 0.004; 56.3% vs 43.7%, *P* = 0.001) by univariate analysis. Age, gender, and VAT, not BMI, were independently associated with difficult colonoscopy by multivariate analysis (OR (95% CI), *P*: 0.964 (0.942, 0.985), 0.001; 1.845 (1.101, 3.091), 0.020; 2.347 (1.395, 3.951), 0.001). Subgroup analysis by gender also showed VAT as the best predictor for both genders.

**Conclusion:**

Difficult colonoscopy was significantly associated with advancing age, female gender and, lower (< 75 cm^2^) or higher (150 cm^2^ ≤) VAT. These subjects may benefit from having complete and more comfortable colonoscopy examinations by using the small-caliber scope rather than the standard scope.

## Introduction

Colonoscopy is a sensitive and popular modality for colon cancer screening and direct diagnosis. The US Multi-Society Task Force on Colorectal Cancer recommends that cecal intubation rate (CIR) is one of many quality indicators of colonoscopy, including adenoma detection rate, bowel preparation, withdrawal time, and sedation practice [1, 2]. The success in colonoscopy mainly depends on subjects, colonoscopists, and the type of colonoscope, and incomplete colonoscopies occur in up to 10% of patients [1, 3]. Thus, it is very important to identify the potentially difficult cases before colonoscopy and change the type of scope, potentially facilitating cecal intubation after procedural failure. There have been many reports demonstrating a variety of factors associated with incomplete colonoscopy or difficult colonoscopy with long cecal intubation time (CIT) (e.g., 10 minutes < CIT), including subjects’ physiques, BMI, bowel preparation, sedation practice, age, gender, previous abdominal or pelvic surgery, and severe diverticular disease [3–12]. In contrast, there is only limited data on the effect of visceral adipose tissue (VAT) on ease of colonoscopy.

The aim of this retrospective study was to analyze the factors related to difficult colonoscopy under conscious sedation and demonstrate the clinical utility of a long small-caliber colonoscope as rescue by using the data from a large number of subjects who underwent general health check-ups.

## Materials and methods

### Study population

We retrospectively searched the electronic clinical databases for consecutive 1036 cases who underwent regular health check-ups, including colonoscopy, over a 12-month period (April 2015 to March 2016). These cases were the same as those reported previously [13]. Colonoscopes used were 260 series variable stiffness instruments (PCF-Q260AI and PCF-PQ260L/I; Olympus Medical System. Tokyo, Japan). The former was used as the standard colonoscope, and the latter was also used as rescue, following incomplete colonoscopy with the standard scope. The PCF-PQ260L scope (outer diameter of 9.2 mm, working length of 1,680 mm) has a passive-bending function, whereby a secondary bending part bends passively and is extremely flexible at the angulated portion of the colon. At our center, all subjects received 2 liters of polyethylene glycol electrolyte solution with a high dose of ascorbic acid (MoviPrep^Ⓡ^, EA Pharma Co, Tokyo, JPN) [14] and 1 liter of clear fluid, according to the manufacture’s instruction. The following data were extracted from subjects’ medical records: age, gender, medical history, physique including BMI and VAT, bowel preparation quality, sedation practice, results of colonoscopy, and pain during colonoscopy intubation evaluated by each colonoscopist. The computed tomography (CT) image of intra-abdominal VAT was obtained for subjects who underwent health check-up with a single cross-sectional scan at the level of the umbilicus, immediately after the non-enhanced chest CT imaging routinely used to screen for chest lesions. The segmentation of the abdominal subcutaneous adipose tissue (SAT) and VAT areas was determined using automated AZE Virtual Place software (AZE Inc., Tokyo, Japan), as the reported technique [15], as previously standardized and validated. The process of estimating abdominal adipose tissue area was optimized by having two experienced radiologists manually removing residual fat-containing tissues. Intubation time was defined as the time taken to intubate the scope from the anus to the cecum, and recorded as the nearest whole time one-minute units. Bowel preparation quality was defined as excellent, good, fair, poor, or inadequate, according to a 5-point categorical scale [16] and the first 3 categories were considered acceptable preparation. Pain assessment by each colonoscopist was defined as none, weak, or strong when additional sedation was given.

The Institutional Review Board of Keio University Hospital approved this retrospective, observational cohort study and the requirement to obtain informed consent was waived (**IRB No. 20160081**).

### Statistical analysis

Baseline data are expressed as the means with standard deviation (SD). *P*-values < 0.05 were considered statistically significant. The statistical difference between the “Easy colonoscopy (CS)” or “Difficult CS” groups was determined by using the Mann-Whitney *U*-test for continuous data and the *χ*^2^ test for the distribution of categorical data. Analysis of variance and *t* tests were also conducted when appropriate for stratified analyses. They included individual subjects and colonoscopy characteristics such as age, gender, BMI, VAT, history of abdominal or pelvic surgery, colonic diverticulosis, and bowel preparation quality, if available. Collinearities among predictor variables were evaluated with Pearson’s product moment and Spearman’s rank correlation coefficients. Only variables with *P*-values of < 0.05, evaluated by univariate analysis, were entered into multivariate logistic regression analysis (likelihood ratio test) to identify significant predictors that were independently related to the risk of difficult colonoscopy, adjusted for the effect of each other. Adjusted odds ratios (OR) and 95% confidence intervals (CI) were estimated. The Hosmer-Lemeshow goodness-of-fit test was used to determine how well the data fit the model and predictive accuracy was calculated. We also performed a multivariate analysis when obesity indices (BMI and VAT) were considered separately. All statistical analyses were performed using SPSS software program (SPSS version 24; SPSS, Inc, Tokyo, JPN).

## Results

### Subject and colonoscopy characteristics

**Fig 1:**
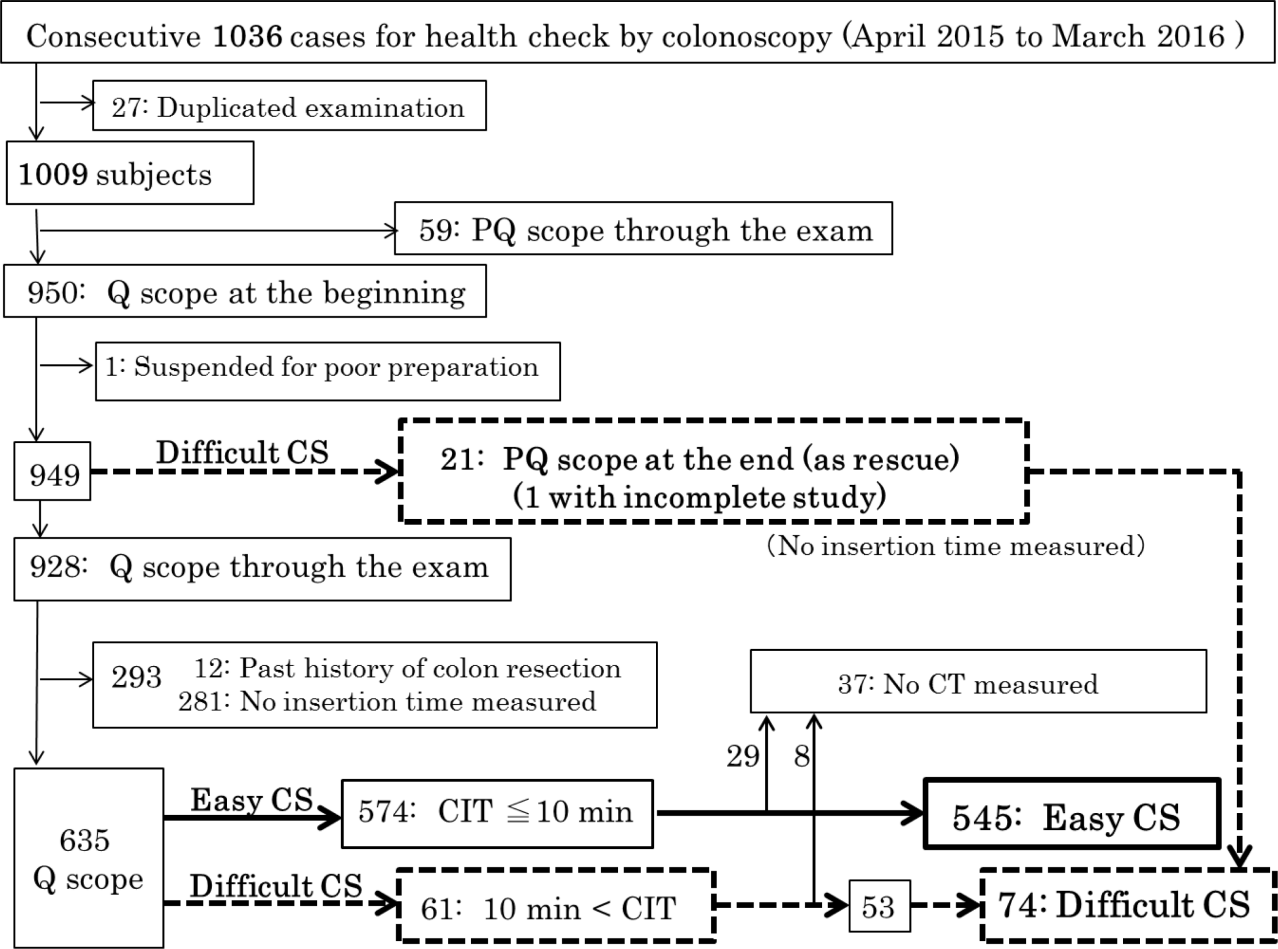
Flow chart of the present study. Flowchart for the selection of study subjects to investigate the factors related to difficult colonoscopy and the clinical usefulness of PQ scope as rescue. CIT: cecal intubation time; CT: computed tomography.

As shown in **Fig 1**, 27 cases who underwent colonoscopy two times within one year were excluded from the 1036. This left 1009 subjects subjected to further analysis. To analyze the factors related to difficult colonoscopy, we removed 59 subjects who underwent colonoscopy by PQ scope and one for poor preparation. This left a total of 949 subjects, including 21 subjects who had reinsertion by PQ scope as rescue in exchange of Q scope, and 928 who completed the examination by Q scope only. Then, 293 subjects were excluded, including 12 with past histories of colon resection and 281 where CIT was not measured. Finally, from the 574 subjects with CIT ≦ 10 min and 61 with CIT > 10 min each, 29 and 8 subjects without CTs were further excluded in order to investigate the relationship between difficult colonoscopy and VAT. Consequently, a total of 619 subjects were included in this study and they were divided into 2 groups: 545 subjects were classified as the “Easy CS” group and 74 (21 in addition with 53) were classified as the “Difficult CS” group. Those in the difficult CS group had colonoscopies where the CIT was > 10 min, or incomplete colonoscopies by the standard scope. Consequently, the “Difficult CS” group comprised 12.0% of the 619 examinations.

Characteristics of the 619 subjects and colonoscopies are summarized in **Table 1**. The median age was 58.0 ± 11.3 years and 446 subjects were males (72.1%). The average of CIT was 6.1 ± 3.8 (1–40) min. No inadequate bowel preparation quality was found and nearly all of bowel preparation qualities were acceptable (excellent, good, or fair, 97.2%). All 619 subjects had undertaken colonoscopy under conscious sedation by administration of an intravenous opiate alone, benzodiazepine alone, or both, and the colonoscopists evaluated that 18.2% of the subjects complained of weak or strong pain. All colonoscopies were performed by the colonoscopists who had > 10 years’ experience. Significantly, difficult CS (n = 53) had longer CIT than easy CS (n = 545) (15.3 ± 5.4 vs 5.2 ± 2.1, *P* = 0.00) and the subjects who underwent difficult CS (n = 64) complained of more pain (weak or strong) during colonoscopy intubation than those who underwent easy CS (n = 492) (50.0% vs 13.6%, *P* = 0.00).

**Table 1:** Summary of the results: Subjects and colonosocpy characteristics.

**Subjects (n = 619)**		
**Age (years)**	n = 619	58.0 ± 11.3 (30–89 years)
**Gender (M, F)**	n = 446, 173 (/619)	72.1%, 27.9%
**Past abdominal surgery (yes, no)**	n = 117, 502 (/619)	18.9%, 81.1%
**Past pelvic surgery (yes, no)**	n = 24, 595 (/619)	3.9%, 96.1%
**Colonoscopy (n = 619)**		
**CIT**	n = 598	6.1 ± 3.8 (1–40 min)
**Bowel prep quality**	n = 546, 16, 0 (/562)	97.2, 2.8, 0%
**(excellent, good, fair; poor; inadequate)**		
**Sedation practice**	n = 465, 3, 151, 0 (/619)	75.1, 0.5, 24.4, 0%
**(opiate, benzodiazepine, both, none)**		
**Pain evaluated by the colonoscopists**	n = 455, 83, 18 (/556)	81.8%, 14.9%, 3.3%
**(none, weak, strong)**		
**Diverticulosis (yes, no)**	n = 197, 422 (/619)	31.8%, 68.2%
**Colonoscopist experience level**	n = 619, 0 (/619)	100%, 0%
**(over 10 years, under 10 years)**		

CIT: cecal intubation time

### Reasons for incomplete colonoscopy using the standard scope

Among 21 subjects who had reinsertion by PQ scope as rescue, in exchange of the Q scope, 20 subjects could complete the examination. One was unable to complete the examination because of an adhesion, resulting in incomplete intubation to the mid-ascending colon. In this case, the prior colonoscopy was discontinued before it reached the sigmoid-descending junction due to the post-operative adhesion. Thus, CIR increased from 97.9% to 99.9% (1007/1008). The main reasons for incomplete colonoscopy by the standard Q scope are presented in **Table 2**. Eight (38.1%) were discontinued for tortuosity when the colonoscope reached the left hemicolon, including six (28.6%) attributed to the sigmoid-descending junction, and six (28.6%) attributed to a redundant colon when the colonoscope reached the right hemicolon. Four (19%) were discontinued due to continuous pain, even after additional opiate administration.

**Table 2:** Reasons for incomplete colonoscopy by the standard Q scope (n = 21).

	Total	Males	Females
**Redundancy**	6 (28.6%)	4	2
**(loop formation, elongation)**			
**Tortuosity**	8 (38.1%)	5	3
**(splenic flexture)**	1	1	0
**(sigmoid-descending junction)**	6	4	2
**(rectal-sigmoid junction)**	1	0	1
**Fixation (adhesion due to post surgery)**	3 (14.3%)	1	2^✽^
**Pain**	4 (19%)	2	2

^✽^including one with incomplete study.

### Distribution of easy CS, difficult CS, and CIT, stratified by BMI and VAT

The upper parts of **Tables 3** and **4** present the subject distribution in the two groups (easy CS and difficult CS), and CIT, stratified into 4 or 5 categories for BMI or VAT. The criteria for obesity was as per WHO classification of BMI into 4 categories (BMI < 18.5 kg/m^2^: underweight; 18.5 kg/m^2^ ≤ BMI < 24.9 kg/m^2^: healthy weight; 25.0 kg/m^2^ ≤ BMI < 29.9 kg/m^2^: overweight; 30 kg/m^2^ ≤ BMI: obesity) [17], while the Japanese criteria for ‘obesity disease’ classifies VAT into 2 categories (normal: VAT < 100 cm^2^; 100 cm^2^ ≤ VAT: visceral fat obesity) [18]. As for BMI, the underweight category included nearly 20% of subjects with difficult CS, while the other 3 categories, corresponding to the healthy weight, the overweight and the obesity categories, accounted for nearly 10% of the difficult CS. Likewise, the mean CIT was longest in the underweight category, compared with that in the other 3 categories where CIT was approximately 6 minutes. Therefore, when we divided 619 subjects into 2 new categories with a BMI of 21 kg/m^2^ as a cut-off point, as shown in the lower part of **Table 3**, the distribution of easy CS, difficult CS, and CIT were significantly associated with the 2 new categories (*P* = 0.004, *P* = 0.003). On the contrary, the third category (100 cm^2^ ≤ VAT < 150 cm^2^) had the lowest percentage of subjects with difficult CS (8.6%) and the shortest CIT (5.4 ± 3.1 minutes), compared with the other 4 categories (upper panel of **Table 4**). Therefore, we divided all the subjects into 3 new categories (VAT < 75 cm^2^, 75 cm^2^ ≤ VAT < 150 cm^2^, 150 cm^2^ ≤ VAT) and calculated the natural logarithm of their odds ratios (0, 0.38, and 0.02, respectively), which showed neither a monotonous increase nor monotonous decrease. Accordingly, when the first category (VAT < 75 cm^2^) was combined with the third category (150 cm^2^ ≤ VAT), and compared with the second category (75 cm^2^ ≤ VAT < 150 cm^2^), the distribution of easy CS, difficult CS, and CIT were significantly associated with the 2 new categories (*P* = 0.001, *P* = 0.001) (lower part of **Table 4**).

**Table 3:** Easy CS, difficult CS and CIT, stratified by BMI.

A					
BMI(kg/m^2^)	n (%)	Easy CS	Difficult CS	CIT	
		n (%)	n (%)	n	
**Category 1–4**	619 (100)	545 (88.0)	74 (12.0)	598	Mean (S.D.)
**1. BMI < 18.5**	24 (3.9)	19 (79.8)	5 (20.8)	23	(7.5)
**2. BMI < 25**	416 (67.2)	364 (87.5)	52 (12.5)	399	(3.7)
**3. BMI < 30**	160 (25.8)	145 (90.6)	15 (9.4)	157	(3.4)
**4. 30 ≤ BMI**	19 (3.1)	17 (89.5)	2 (10.5)	19	6.5 (3.5)
**B**					
**New Category 1, 2**	619 (100)	545 (88.0)	74 (12.0)	598	Mean (S.D.)
**1. BMI < 21**	130 (21.0)	105 (80.8)	25 (19.2)	121	7.3 (5.1)
**2. 21 ≤ BMI**	489 (79.0)	440 (90.0)	49 (10.0)	477	5.8 (3.4)

CS: colonoscopy; CIT: cecal intubation time.

**Table 4:** Easy CS, difficult CS and CIT, stratified by VAT.

A						
VAT (cm^2^)	n (%)	Easy CS	Difficult CS		CIT	
		n (%)	n (%)		n	
**Category 1–5**	619 (100)	545 (88.0)	74 (12.0)		598	Mean (S.D.)
**1. VAT < 50**	89 (14.4)	75 (84.3)	14 (15.7)		84	7.1 (5.2)
**2. VAT < 100**	239 (38.6)	211 (88.3)	28 (11.7)		232	6.2 (3.5)
**3. VAT < 150**	198 (32.0)	181 (91.4)	17 (8.6)		193	5.4 (3.1)
**4. VAT < 200**	69 (11.1)	59 (85.5)	10 (14.5)		67	6.2 (4.1)
**5. 200 ≤ VAT**	24 (3.9)	19 (79.2)	5 (20.8)		22	7.8 (5.4)
**B**						
**New Category 1, 2**	619 (100)	545 (88.0)	74 (12.0)	598		Mean (S.D.)
**1. VAT < 75 or 150 ≤ VAT**	(46.2)	238 (83.2)	48 (16.8)	272		6.9 (4.5)
**2. 75 ≤ VAT < 150**	333 (53.8)	307 (92.2)	26 (7.8)	326		5.5 (3.1)

CS: colonoscopy; CIT: cecal intubation time; VAT: visceral adipose tissue.

### Univariate and multivariate analysis of the factors related to difficult colonoscopy

Associations between subject-related, procedure-related factors and difficult colonoscopy are summarized in **Table 5**. Significantly, the subjects who underwent difficult CS (n = 74) were older than those who underwent easy CS (n = 545) (61.6 ± 12.8 vs 57.5 ± 11.0, *P* = 0.003). The subjects with easy CS were mostly male (73.9% vs 58.1%, *P* = 0.004) and taller (167.2 ± 8.1 vs 165.2 ± 9.4, *P* = 0.047), compared to those subjects with difficult CS. Moreover, higher BMI (21 kg/m^2^ ≤ BMI) and intermediate VAT (75 cm^2^ ≤ VAT < 150 cm^2^) were significantly associated with easy CS (80.7% vs 19.3%, *P* = 0.004; 56.3% vs 43.7%, *P* = 0.001). Body weight, waist circumference, history of abdominal or pelvic surgery, colonic diverticulosis, and bowel preparation had no impact on the ease of colonoscopy.

**Table 5:** Univariate analysis of the factors related to difficult colonoscopy.

Factors		n	Average (S.D) or Number (%)	*P* -value
**Age** (year)	**Easy CS**	545	57.5 (11.0)	**0.003**
	**Difficult CS**	74	61.6 (12.8)	
**Gender** (Male)	**Easy CS**	545	403 (73.9)	**0.004**
	**Difficult CS**	74	43 (58.1)	
**Height** (cm)	**Easy CS**	545	167.2 (8.1)	**0.047**
	**Difficult CS**	74	165.2 (9.4)	
**Body weight** (kg)	**Easy CS**	545	67.5 (31.6)	0.278
	**Difficult CS**	74	63.4 (17.0)	
**BMI** (kg/m^2^)	**Easy CS**	545	105 (19.3), 440 (80.7)	**0.004**
(BMI < 21, 21 ≤ BMI)	**Difficult CS**	74	25 (33.8), 49 (66.2)	
**VAT** (cm^2^)	**Easy CS**	545	238 (43.7), 307 (56.3)	**0.001**
(VAT < 75 or 150 ≤ VAT,				
75 ≤ VAT <150)	**Difficult CS**	74	48 (64.9), 26 (35.1)	
**Waist** (cm)	**Easy CS**	545	84.1 (9.3)	0.494
	**Difficult CS**	74	83.0 (13.4)	
**History of abdominal**	**Easy CS**	545	101 (18.5)	0.524
**surgery** (yes)	**Difficult CS**	74	16 (21.6)	
**History of pelvic surgery**	**Easy CS**	545	19 (3.5)	0.147
(yes)	**Difficult CS**	74	5 (6.8)	
**Colonic diverticulosis**	**Easy CS**	545	178 (32.7)	0.215
(yes)	**Difficult CS**	74	19 (25.7)	
**Bowel prep quality**	**Easy CS**	499	485 (97.2)	0.303
(Good to Fair)	**Difficult CS**	64	61 (95.3)	

CS: colonoscopy; VAT: visceral adipose tissue.

Next, we confirmed that none of the predictor variables were highly correlated (r ≤ 0.622). Therefore, when BMI and VAT were considered simultaneously by multivariate analysis, age, gender, and VAT were independently associated with difficult colonoscopy (OR (95% CI), *P*: 0.964 (0.942, 0.985), 0.001; 1.845 (1.101, 3.091), 0.020; 2.347 (1.395, 3.951), 0.001) (**Table 6**). The Hosmer-Lemeshow goodness-of-fit test indicated that this model was a good fit to the data (*P* = 0.75) with high predictive accuracy (88%). To analyze the obesity indices (BMI and VAT) separately, when age, gender, height and BMI were included in multivariate analysis, age (OR (95% CI), *P*: 0.967 (0.946, 0.989), 0.003) and BMI (2.472 (1.462, 4.180), 0.001) remained independently associated with difficult colonoscopy (the 4th and 5th column in **S1 Table**). But the Hosmer-Lemeshow test results were not as high as when the factors were analyzed simultaneously (*P* = 0.407 vs *P* = 0.75). On the contrary, when age, gender, height and VAT were included in a multivariate analysis, age (OR (95% CI), *P*: 0.962 (0.941, 0.984), 0.001), gender (1.720 (1.003, 2.948), 0.049) and VAT (1.879 (1.093, 3.230), 0.022) were independently associated with difficult colonoscopy (the second and third column in **S1 Table**) and this model was also a good fit to the data (*P* = 0.75).

**Table 6:** Multivariate analysis of the predictors for difficult colonoscopy when the obesity indices were considered simultaneously (overall).

Predictors	Odds Ratio (95% CI)	*P*-value
**Age** (year)	**0.964** (0.942, 0.985)	**0.001**
**Gender**	**1.845** (1.101, 3.091)	**0.020**
**Height** (cm)		0.187
**BMI** (kg/m^2^)		0.342
(BMI < 21; 21 ≤ BMI)		
**VAT** (cm^2^)	**2.347** (1.395, 3.951)	**0.001**
(VAT < 75 or 150 ≤ VAT; 75 ≤ VAT < 150)		

VAT: visceral adipose tissue.

Finally, we performed a sub-analysis, using divided data for males and females. Univariate analysis in males showed that age, BMI and VAT were significantly associated with difficult colonoscopy (*P* < 0.05, respectively). Among these variables, age (OR (95% CI), *P*: 0.906 (0.932, 0.988), 0.006) and VAT (1.926 (1.002, 3.700), 0.049) were significant predictors of difficult colonoscopy in multivariate analysis (**Table 7**). On the other hand, univariate analysis in females showed that age and VAT were significantly associated with difficult colonoscopy (*P* < 0.05, respectively). Among these variables, only VAT (OR (95% CI), *P*: 2.582 (1.044, 6.383), 0.040) was a significant predictor of difficult colonoscopy in multivariate analysis (**Table 8**). Thus, most importantly, VAT was the best predictor for difficult colonoscopy, even in the subgroup analysis by gender.

**Table 7:** Multivariate analysis of the predictors for difficult colonoscopy in males.

Predictors	Odds Ratio (95% CI)	*P*-value
**Age** (year)	**0.960** (0.932, 0.988)	**0.006**
**BMI** (kg/m^2^)		0.054
(BMI < 21; 21 ≤ BMI)		
**VAT** (cm^2^)	**1.926** (1.002, 3.700)	**0.049**
(VAT < 75 or 150 ≤ VAT; 75 ≤ VAT < 150)		

VAT: visceral adipose tissue.

**Table 8:** Multivariate analysis of the predictors for difficult colonoscopy in females.

Predictors	Odds Ratio (95% CI)	*P*-value
**Age** (year)		0.083
**BMI** (kg/m^2^)	N/A	N/A
(BMI < 21; 21 ≤ BMI)		
**VAT** (cm^2^)	**2.582** (1.044, 6.383)	**0.040**
(VAT < 75 or 150 ≤ VAT; 75 ≤ VAT < 150)		

VAT: visceral adipose tissue; N/A: not available.

## Discussion

The US Multi-Society Task Force recommends that CIR should be above 95% for screening colonoscopy [1]. In the present study, CIR reached nearly 100% by the usage of PQ scope as rescue and compared favorably with the minimum standard (≥ 90%) and target (≥ 97%) set forth by the Bowel Cancer Screening Programme [2]. The main reasons for discontinuation by the standard colonoscope were tortuosity in the left hemicolon (n = 8), redundancy in the right hemicolon (n = 6), pain (n = 4) and adhesions (n = 3). Cecum intubation following incomplete colonoscopy was achieved by the rescue colonoscope in 95% cases (20/21), except one with adhesions, resulting in a redundant colon. Moreover, Sato et al. demonstrated that this small-caliber colonoscope reduced pain significantly in female patients during a randomized control trial [19]. In our study, all rescue colonoscopies were completed without additional sedative agents. Although the PQ scope may have the disadvantage of making excessive looping in the proximal colon because of its flexibility, particularly in subjects with a redundant colon, the longer (1,680 mm) and smaller (9.2 mm) PQ-260L scope (compared to the standard scope of 1,330 mm length and 12.0 mm width) could overcome longer colon length and/or more acute angles at the flexure due to redundancy, tortuosity, or fixation.

Advancing age and female gender were reported to be associated with lower CIR and longer CIT [3–8]. The colons of older subjects may tend to be longer, resulting in increased redundancy and loop formation [20] and female colon could be more angulated due to smaller abdominal cavity, or deeper and narrower pelvic space. Additionally, decreased abdominal wall musculature in females could neither provide enough resistance, nor help prevent looping.

Some groups reported that abdominal and pelvic surgeries, including hysterectomies in females, were associated with incomplete colonoscopy [4, 9–11], but the present study showed no significant influence on ease of colonoscopy. This may be a consequence of the relative small number of females (n = 173) in our study.

It is well known that lower BMI is associated with longer CIT and more pain during scope insertion possibly due to sharper angulation of the sigmoid colon and difficulty straightening the scope [6, 8, 9, 12]. Visceral fat may help prevent looping formation, as those with higher BMI may have more fat, so that the colonoscope could reach the cecum more easily [6]. However, only a few recent studies have assessed the effect of VAT on difficult colonoscopy [21–23]. In the present study, lower BMI (BMI < 21 kg/m^2^) was related to longer CIT during univariate analysis, but its effect disappeared in multivariate analysis, when another obesity index (VAT) was considered simultaneously. Interestingly, VAT, unlike BMI, could have a dual effect on difficult colonoscopy. Lower VAT (VAT < 75 cm^2^) had impact on both the ease of colonoscopy and longer CIT. This result is consistent with former reports demonstrating that lower VAT was significantly associated with longer CIT [21–23], since higher VAT might provide resistance and help prevent looping. However, to our surprise, the multivariate analysis also demonstrated that higher VAT (150 cm^2^ ≤ VAT) caused difficult colonoscopy and longer CIT. One of the plausible explanations may be that the intubation of the colonoscope can be more difficult in subjects with large abdomens, requiring a longer intubation time when there is a need for abdominal pressure and/or change of body position. We sometimes experience such cases in clinical practice, where abdominal pressure is frequently ineffective, especially for male subjects having larger waist circumference and, possibly higher SAT and VAT, as males tend to have more fat around the belly than females who tend to carry fat on the buttocks and thighs. Moreover, when analyzed with stratification by gender, VAT had the highest OR, in both genders. On the contrary, BMI had no impact on difficult colonoscopy by multivariate analysis. This may be partially because BMI is affected by the weight of both adipose tissue and muscle tissue, and as the amount of muscle tissue increases, loop formation could be prevented in the same way as abdominal pressure from outside the body.

The strengths of this study are that all of the subjects in the analyzed population (n = 619) underwent screening colonoscopy under conscious sedation by experienced colonoscopists, with a high percentage (97.2%) of acceptable bowel preparations, which exceeded the target (≥ 95%) set by the Bowel Cancer Screening Programme [2]. There was no sedation bias relative to factors that predicted pain and difficulty. Thus, this study design could minimize the effects of other factors (sedation, bowel preparation, technical ability by colonoscopists) aside from subject-related factors. This study had some limitations, including its retrospective and single center design. The mean body size of Japanese subjects tends to be smaller than that of Western subjects, limiting the generalization of our findings regarding the cut-off point of VAT.

In conclusion, subject-related factors associated with difficult colonoscopy included advancing age, female gender and, especially, lower (< 75 cm^2^) or higher (150 cm^2^ ≤) VAT. Subgroup analysis by gender also showed VAT as the best predictor for both genders. This study also suggest that these subjects may benefit from having complete and more comfortable colonoscopy examinations under conscious sedation by using the long small-caliber scope, instead of the standard scope.

## Supporting information

S1 Table Multivariate analysis of the predictors for difficult colonoscopy when the obesity indices were considered separately (overall). (DOCX)
