# Supplementary material for: The impact of visceral adipose tissue as best predictor for difficult colonoscopy and the clinical utility of a long small-caliber scope as rescue
Source: PLoS One. 2017 Dec 21;12(12):e0189817. doi: 10.1371/journal.pone.0189817 (PMC5739452; doi:10.1371/journal.pone.0189817)
Supplement: S1 Table — (DOCX) [file pone.0189817.s001.docx]

**S1 Table. Multivariate analysis of the predictors for difficult colonoscopy when the**

**obesity indices were considered separately (overall).**

| **Predictors** | **OR (95%CI)** | ***P*** | **OR (95%CI)** | ***P*** |
| --- | --- | --- | --- | --- |
| **Age** (year) | **0.962** (0.941, 0.984) | **0.001** | **0.967** (0.946, 0.989) | **0.003** |
| **Gender** | **1.720** (1.003, 2.948) | **0.049** |  | 0.077 |
| **Height** (cm) | | 0.908 |  | 0.208 |
| **BMI** (kg/m^2^)  (BMI < 21; 21 ≤ BMI) | N/A | N/A | **2.472** (1.462, 4.180) | **0.001** |
| **VAT** (cm2)  (VAT < 75 or 150 ≤ VAT; 75 ≤ VAT < 150) | **1.879** (1.093, 3.230) | **0.022** | N/A | N/A |

**VAT: visceral adipose tissue; N/A: not available**
